# Dynamic Expression Profile of Follicles at Different Stages in High- and Low-Production Laying Hens

**DOI:** 10.3390/genes15010040

**Published:** 2023-12-26

**Authors:** Lan Yang, Xuewei Fan, Kaiyuan Tian, Sensen Yan, Chunhong Xu, Yixiang Tian, Chengpeng Xiao, Xintao Jia, Junlai Shi, Ying Bai, Wenting Li

**Affiliations:** 1 College of Animal Science and Technology, Henan Agricultural University, Zhengzhou 450001, China; yanglan_926@163.com (L.Y.); fanxuewei_0910@163.com (X.F.); t18337139678@163.com (K.T.); m18736824024@163.com (S.Y.); xch17806284889@163.com (C.X.); 18790301745@163.com (C.X.); jxt1070@gmail.com (X.J.); sjl15638637310@163.com (J.S.); 2 The Shennong Laboratory, Zhengzhou 450046, China; 3 Henan Institute of Science and Technology, College of Animal Science and Veterinary Medicine, Xinxiang 453003, China; t13071027821@163.com; 4 School of Life Science and Food Engineering, Hebei University of Engineering, Handan 056038, China

**Keywords:** Taihang chicken, clutch traits, follicular development, transcriptome

## Abstract

Improving the efficiency of hens and extending the egg-laying cycle require maintaining high egg production in the later stages. The ovarian follicles, as the primary functional units for ovarian development and oocyte maturation, play a crucial role in regulating the continuous ovulation of hens. The egg production rate of laying hens is mostly affected by proper follicle growth and ovulation in the ovaries. The objective of this study was to identify the key genes and signaling pathways involved in the development of ovarian follicles in Taihang hens through transcriptome screening. In this study, RNA sequencing was used to compare and analyze the transcriptomes of ovarian follicles at four developmental stages: small white follicles (SWF), small yellow follicles (SYF), F5 follicles, and F2 follicles, from two groups: the high continual production group (H-Group) and the low continual production group (L-Group). A total of 24 cDNA libraries were constructed, and significant differential expression of 96, 199, 591, and 314 mRNAs was detected in the SWF, SYF, F5, and F2 follicles of the H and L groups, respectively. Based on the results of GO and KEGG enrichment analyses, each stage of follicle growth possesses distinct molecular genetic features, which have important effects on follicle development and significantly promote the formation of continuous production traits through the biosynthesis of steroid hormones, cytokine–cytokine receptor interaction, and neuroactive ligand–receptor interaction. Additionally, through STEM analysis, we identified 59 DEGs, including *ZP4*, *KCNH1*, *IGFs*, *HMGA2*, and *CDH1*, potentially associated with follicular development within four significant modules. This study represents the first transcriptome investigation of follicles in hens with high and low egg-producing characteristics at four crucial developmental stages. These findings provide important molecular evidence for understanding the regulation of follicular development and its variations.

## 1. Introduction

As an important economic product in the poultry industry, eggs are one of the primary sources of animal protein intake. Through long-term breeding, high-yield production, and continuous advancements in feed and management practices, laying hens can lay nearly one egg per day throughout their laying cycle. The normal development of follicles at each stage in the ovary and their smooth transition to the next cycle are crucial factors that impact the quantity of egg production. High-yielding layers exhibit superior follicular structures compared to low-yielding ones [1]. Ovarian follicle development is a dynamic and complex process. When a hen reaches sexual maturity, its ovarian activity intensifies, and oocytes start forming follicles of various sizes, resembling a cluster of grapes with accumulated nutrients. Avian follicle development is characterized by recruitment, selection, and dominance, establishing a strict grading system. Based on their color and diameter, follicles can be classified as prehierarchical follicles, including small white follicles (SWF, 1–4 mm in diameter), large white follicles (LWF, 4–6 mm in diameter), small yellow follicles (SYF, 6–8 mm in diameter), and hierarchical follicles (diameter larger than 8 mm, classified as F5 to F1) [2]. Primordial follicles develop into a pool of small yellow follicles. Only one dominant follicle is selected to enter the hierarchical follicles. The selected follicle undergoes rapid yolk accumulation, rapidly increasing its diameter, ultimately leading to ovulation [2].

Several studies have reported important mechanisms and genes that affect egg production in poultry. An analysis of the transcriptome of ovarian tissues in Jinghai Yellow chickens with varying levels of egg production identified 305 differentially expressed genes (DEGs) [3]. In both groups, seven genes involved in calcium signaling pathways, cell adhesion, and cytokine–cytokine receptor interaction were up-regulated, suggesting a potentially positive impact of these three pathways on egg production in chickens. Furthermore, transcriptome analysis of SWF, SYF, and LYF at different stages of egg production in Nandan Yao chickens identified key genes such as *COL4A2*, *COL12A1*, *ELN*, *FBN2*, *ALB*, and *MMP10*. These genes may regulate egg production through pathways involving extracellular matrix, extracellular matrix-receptor interaction, collagen-rich extracellular matrix, and collagen trimer formation [4]. Similar transcriptome analyses of ovarian follicles have also been conducted in ducks. The high-egg-production group exhibited increased activity in several important functional pathways compared to the low-egg-production group. These pathways include neuroactive ligand–receptor interaction, circadian rhythm, steroid biosynthesis, fatty acid biosynthesis, calcium signaling pathway, endocrine regulation, and calcium reabsorption regulated by other factors. Additionally, some differentially expressed genes, such as *MC5R*, *APOD*, *ORAI1*, and *DYRK4*, showed higher levels of activity in the ovaries of high-egg-production ducks when compared to those with low egg production [5].

The follicle, which serves as the fundamental functional unit of oogenesis and oocyte formation, plays a crucial role in regulating female reproductive activity. The occurrence, atresia, and maturation of follicles are closely associated with subtle changes in follicle function at different stages. Undoubtedly, follicles at different developmental stages offer ideal targets for exploring the molecular mechanisms of egg production. Moreover, the dynamic expression profiles of follicles at different stages are essential for comprehending the molecular and genetic mechanisms underlying egg production. Therefore, we employed RNA-seq technology to compare the dynamic expression profiles of follicles at different developmental stages between high and low-egg-producing hens. We aimed to identify differentially expressed genes during the dynamics of follicle development and uncover the associated biological processes and pathways that regulate the development of growing follicles. These approaches provided insights into the regulation of maturation and changes in follicle development in the two groups of hens.

## 2. Materials and Methods

### 2.1. Ethics Approval

The study adhered to the Regulations for the Administration of Affairs Concerning Experimental Animals (Ministry of Science and Technology, Beijing, China, 2004) for conducting animal experiments and nursing. Furthermore, the experimental protocol received approval from the Animal Care and Use Committee (IACUC) of Henan Agricultural University, with the assigned approval number 11-0085.

### 2.2. Hen Selection and Follicular Collection

In this experiment, we collected egg production records from 704 Taihang chickens aged 32 to 50 weeks. Egg collection was carried out daily, starting at 2 PM, and individual egg production records were meticulously documented. To meet the nutritional requirements of Taihang chickens for egg production, we fed them 3–4 times a day with Zhengda 524 laying hens formula feed. The lighting duration was set at 16 h per day, with a lighting intensity ranging from 20 lux to 30 lux. The humidity inside the housing was maintained between 40% and 60%. Then, several egg production characteristics were statistically summarized, including the number of clutch cycles, average clutch length (CL), length of large clutch, average resting length, average clutch cycle, and average clutch intensity. The group was divided into high- and low-laying groups based on egg production. Three healthy individuals were randomly selected from each group, and their SWF, SYF, F5, and F2 were collected. The follicles were promptly frozen in liquid nitrogen and stored at −80 °C after separation using medical forceps and scissors.

### 2.3. Total RNA Extraction and cDNA Library Construction

Total RNA was extracted from SWF, SYF, F5, and F2 using the Tiangen Total RNA Extraction Kit, following the provided instructions. First, approximately 200–300 mg of follicular tissue was ground in liquid nitrogen. Then, 1 mL of lysis buffer RZ was added, and the mixture was homogenized using a homogenizer. Next, 200 µL of chloroform was added, and the mixture was vigorously shaken for 15 s. The mixture was then allowed to stand on ice for 3 min to facilitate the separation of liquid phases. Subsequently, the centrifuge tube was placed in a 4 °C centrifuge and spun at 12,000 rpm for 10 min. After that, an equal volume of ethanol (without water) was added and thoroughly mixed, and the resulting liquid was transferred to an adsorption column CR3. The column was then centrifuged at 12,000 rpm for 30 s at 4 °C, and the waste was discarded in the collection tube. To the adsorption column CR3, 500 µL of protein removal solution RD was added, and the mixture was centrifuged at 4 °C and 12,000 rpm for 30 s, followed by the discarding of the waste. Next, 500 µL of wash solution RW was added to the adsorption column CR3, allowing it to sit at room temperature for 2 min, and then centrifuged at 4 °C and 12,000 rpm for 30 s. The waste was discarded, and this step was repeated. The adsorption column was placed in the collection tube and centrifuged at 4 °C and 12,000 rpm for 2 min to remove residual liquid. The adsorption column CR3 was transferred to a new 1.5 mL RNase-free tube, and 20 µL of RNase-free water was added. After a 2-min incubation at room temperature, the mixture was centrifuged at 4 °C and 12,000 rpm for 2 min. RNA concentration was checked using a Thermo NanoDrop 2000 ultra-micro spectrophotometer (OD260/280: 1.8~2.0), while RNA fragment length was determined using the Agilent 2100 system. In this project, qualified samples were selected for mRNA enrichment using magnetic beads with Oligo(dT). A fragmentation buffer was added to break the mRNA into fragments. The first strand of cDNA was synthesized through reverse transcription, using mRNA as a template and 6-bp random primers. The second strand was obtained by adding a buffer, dNTPs, and DNA polymerase I. After purifying the double-stranded cDNA with AMPure XP beads, end repair and ploy A-tail were performed. Finally, the cDNA library was constructed by PCR amplification. The effective concentration of the library was precisely quantified to satisfy high-quality library standards (>4 nM). Subsequently, the libraries were sequenced based on the Illumina sequencing platform (HiSeq 2500).

### 2.4. Sequencing Data Quality Control and Alignment

The raw data was filtered to remove reads containing adapters (Trimmomatic v0.33) and low-quality bases (Q ≤ 20) with a content exceeding 50% (FastQC v 0.11.8). This filtering process ensured the acquisition of high-quality, clean data for subsequent analysis. The clean reads were then mapped to the reference genome (*Gallus gallus*, Galgal5; https://www.ncbi.nlm.nih.gov/assembly/GCF_000002315.4, accessed on 3 December 2020) of chickens using TopHat2 [6]. The reads were assembled into novel transcripts using the Cufflinks software v2.1.1. Subsequently, the Cuffcompare was used to compare these transcripts with a known database to identify unannotated novel genes.

### 2.5. Analysis of Gene Expression

The expression levels of genes were measured using the fragments per kilobase of transcript per million mapped reads (FPKM) method. An FPKM value greater than 1 was set as the threshold [7]. Differential gene expression analysis of mRNA was performed using DEGseq [8], with a significance threshold of a *p*-value < 0.05.

### 2.6. Function Enrichment Analysis

Gene Ontology (GO) and Kyoto Encyclopedia of Genes and Genomes (KEGG) enrichment analysis and functional annotation were processed by KEGG Orthology-Based Annotation System (KOBAS) network software version 3.0 (http://bioinfo.org/kobas, accessed on 12 August 2023) [9]. The statistical test method employed the system’s default hypergeometric and Fisher’s exact tests. Then, the *p*-value < 0.05 was set as the cut-off criterion. The OmicShare tool is a free online data analysis platform for graphic rendering.

### 2.7. Real-Time Quantitative PCR Analysis

Quantitative PCR (qPCR) was employed to assess the transcription levels of nine genes in ovarian follicles, aiming to confirm the accuracy and reliability of the RNA-Seq results. RNA was reverse transcribed into cDNA using the HiScript III RT SuperMix kit (Vazyme) for amplified candidate genes (including *GDF9*, *STAR*, *FSHR,* and *IGF-2*). Primer information is listed in Table 1. The transcription level of each target gene was normalized to the housekeeping gene *GAPDH* using the 2^−ΔΔCt^ method.

## 3. Results

### 3.1. Assessment of Taihang Layer Egg Production Capacity

**Figure 1:**
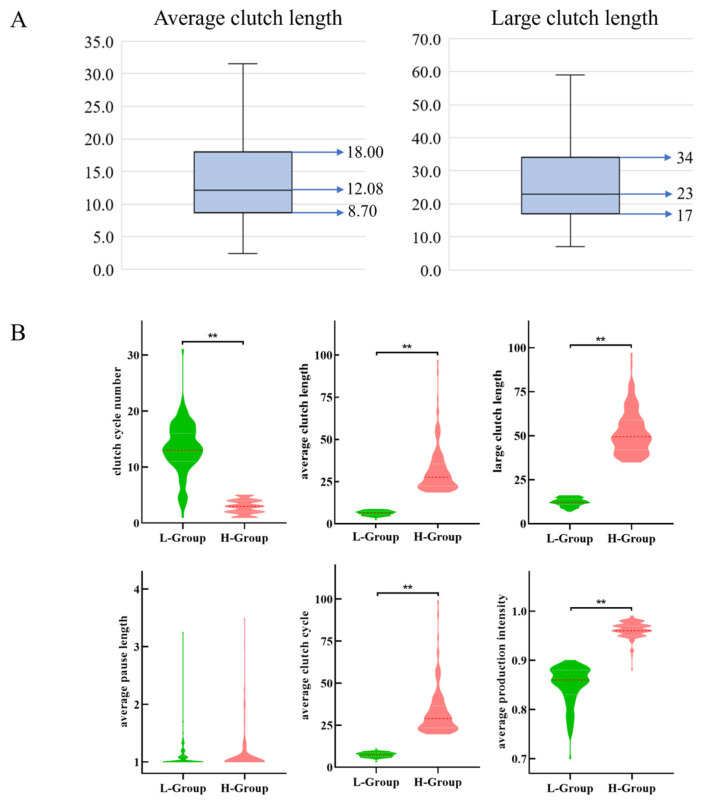
Clutch traits in Taihang chickens. (**A**) Average (**left**) and large (**right**) CL in 704 female chickens. (**B**) Phenotypic analysis of clutch traits in the validated Taihang population with 240 individuals. **, *p* < 0.01.

Egg production records analysis was conducted on 704 Taihang laying hens aged between 33 and 50 weeks. The median average CL was 12.08, 8.7, and 18 days in the lower and upper quartiles. The median of the large CL was 23, 17, and 34 days in the lower and upper quartiles, respectively (Figure 1A). The number of chickens with a large CL over 35 days accounted for 22.9%. Individuals with an average CL below 8.7 days and above 18 days and those with a large CL below 17 days and over 34 days were classified as the low- and high-yield groups, respectively. Finally, there were 118 hens in the low-egg-producing group and 122 in the high-egg-producing group.

Based on the previously announced grouping results, a significance analysis was conducted to validate the feasibility of the clutch characters between the high and low groups. The average CL and clutch intensity showed the most significant differences (*p* < 0.01). Unexpectedly, the average interval length was not significant (Figure 1B). Three hens were randomly selected from the high-egg-producing and low-egg-producing groups for differential expression analysis.

### 3.2. Data Summary of mRNAs in Chicken Follicular

**Figure 2:**
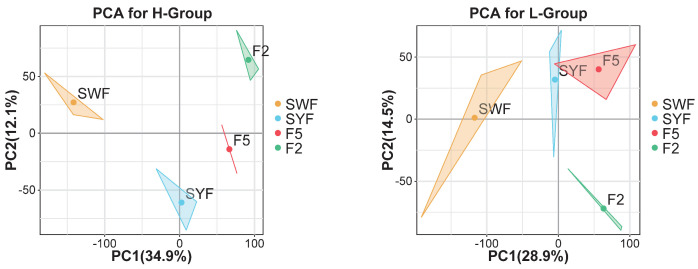
Principal component analysis (PCA) of the H- (**left**) and L- (**right**) groups based on mRNA transcriptomics. Different colors represent different follicles, and dots represent centroids.

To systematically explore the follicular transcriptome during chicken development, granulosa cells were collected from follicles at eight developmental stages, i.e., SWF, SYF, F5, and F2 follicles. These samples underwent high-throughput RNA analyses. A total of 219.52 Gb of raw data was generated. After filtering adaptors and low-quality reads, 213.88 Gb of clean bases were retained, corresponding to an average of ∼8.91 Gb of high-quality reads per sample. The high-quality reads were then mapped to the chicken genome (*G. gallus*-5.0) using Tophat 2.1.0, with a mapping rate ranging from 80.42% to 82.73% (Table S1). Principal component analysis (PCA) was performed using two principal components (PC1 and PC2) to demonstrate the source of variance in the data. The results showed that the follicular samples were collected and separated based on different stages, suggesting that the mRNA was expressed in a stage-specific manner (Figure 2).

### 3.3. Identification of Differentially Expressed mRNAs

To identify DEGs in high- and low-yielding chicken follicular tissues, we compared expression levels between the four developmental stages with a threshold of |FC| > 2 and *p* < 0.05. We determined the DEGs between the H-Group and L-Group within SWF, SYF, F5, and F2 follicles (Table 2). In SWF follicles, 60.4% of the 96 DEGs were up-regulated. In SYF, F5, and F2 follicles, there were 199,591 and 314 differential genes, respectively, with the proportion of up-regulated genes being 16.1%, 8.63%, and 10.51% respectively. Additionally, there were more DEGs in the F5 follicles of the H- and L-Group compared to the other follicles.

**Figure 3:**
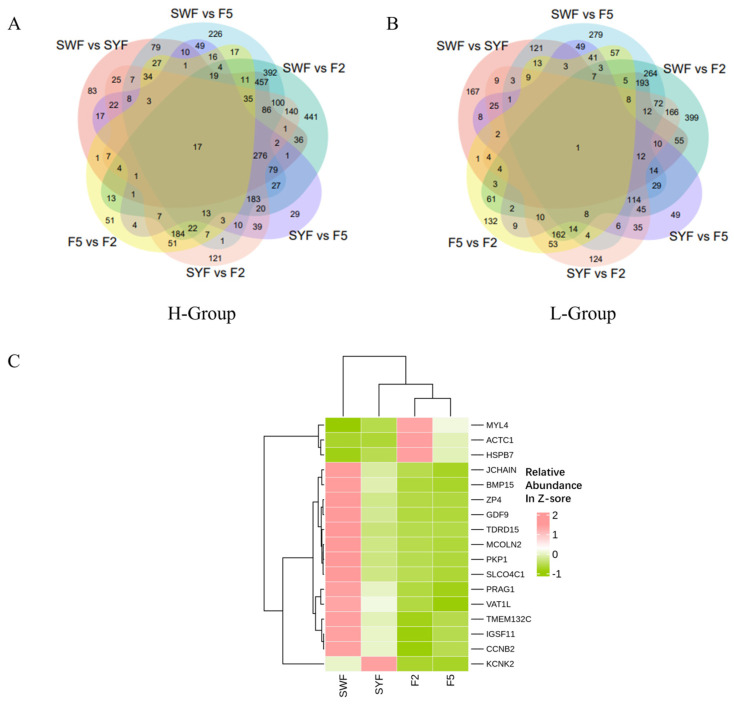
Differentially expressed mRNAs. In the H (**A**) and L (**B**) groups, the Venn diagrams of common differentially expressed mRNAs in each stage. (**C**) Heatmap of 17 genes dynamic expression of H-Group.

Substantial differential expression was observed across the four developmental phases through pairwise comparisons of the six matches; we identified 3520 unique mRNAs in the high group and 2877 unique mRNAs in the low group. In the H-Group, the largest number of DEGs was observed in the SWF versus F2 comparison, followed by SWF versus F5 and SYF versus F2. A similar trend was observed in the low group (Figure 3A; Table S2).

The Venn diagram provides a further description of the expression patterns of DEGs during the four stages of follicle development. The Venn diagram, which includes six groups (SWF vs. SYF, SWF vs. F5, SWF vs. F2, SYF vs. F5, SYF vs. F2, and F5 vs. F2), reveals that the H Group had 8744 DEGs, while the L Group had 6924 DEGs. In the L-Group, only one DEG was consistently expressed across all four developmental stages. This mRNA is *ACTC1*, which encodes α-cardiac actin, a major component of the contractile apparatus found in cardiac muscles. Its presence suggests potential involvement in developing skeletal muscles and vascular endothelial processes.

Interestingly, *ACTC1* is found in cardiac muscle cells and in female reproductive cells [10,11,12]. On the other hand, 17 common DEGs displayed different expression patterns at each follicle development stage, as shown in the heatmap (Figure 3B). The expression levels of *ACTC1*, *MYL4*, and *HSPB7* increased across all stages, while *KCNK2* reached its peak expression at the SYF stage. Conversely, the expression of *IGSF11*, *TMEM132C*, *CCNB2*, and others consistently decreased across all stages. *GDF9*, *BMP15*, *VAT1L*, *ZP4*, *TDRD15*, and five other genes reached their lowest expression point at the F5 stage, indicating their contribution to follicle selection and development to meet the high egg production demand in laying hens.

### 3.4. Functional Clustering of DEGs at Different Growth Follicles

**Figure 4:**
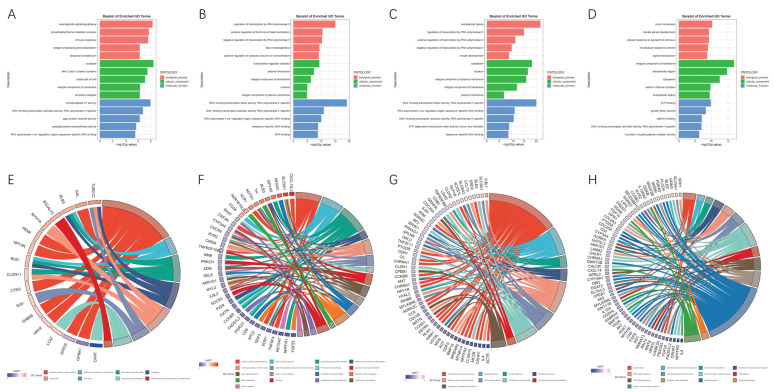
Functional annotation and enrichment analysis of DEGs. (**A**–**D**) Go enrichment analysis bar graphs. (**E**–**H**) KEGG enrichment analysis chord diagram. Enriched pathways (**right**) and differential genes (**left**) are organized by their expression levels.

To characterize the differential expression profiles of follicles in the H-Group and L-Group, we compared mRNAs from each developmental stage. Functional enrichment analysis was performed on the DEGs (Figure 4).

In the SWF follicles, the DEGs and their corresponding functional enrichment analysis are presented in Table S3. A large number of genes were enriched in terms related to synapses, membrane composition, extracellular space, DNA, and RNA activity (Figure 4A). Figure 4E presents the terms associated with KEGG pathways. There were eight relevant KEGG pathways identified, including neuroactive ligand–receptor interaction (gga04080), the intestinal immune network for IgA production (gga04672), cell adhesion molecules (CAMs) (gga04514), phagosome (gga04145), tight junction (gga04530), gap junction (gga04540), glycerophospholipid metabolism (gga00564), and Glycosphingolipid biosynthesis-globo and isoglobo series (gga00603) (Table S3). In total, there were 96 DEGs, with 58 up-regulated and 38 down-regulated genes, implicated in these eight signaling pathways.

The comparison in SYF identified 32 increased and 167 decreased DEGs (Table S2). The functional enrichment analysis results for each DEG are summarized in Figure 4B,F and Table S4. GO terms associated with the downregulated DEGs include cytoplasm, nucleoplasm, plasma membrane, and nucleus. Pathway analysis revealed significant enrichment of these DEGs in aspects such as cytokine–cytokine receptor interaction, the calcium signaling pathway, neuroactive ligand–receptor interaction, and neuroactive ligand–receptor interaction. Five downregulated genes (*FADS1L2*, *BAAT*, *AKR1D1*, *CYP3A4*, and *RETSAT*) showed significant enrichment in pathways associated with the biosynthesis of unsaturated fatty acids, steroid hormone biosynthesis, steroid hormone biosynthesis, and retinol metabolism.

Among these two fertility groups, the F5 stage exhibited the highest number of DEGs (Table 2). Several potentially interesting terms for biological processes were identified, including oocyte development, ovarian follicle development, negative regulation of the apoptosis signaling pathway, and oocyte maturation. The DEGs were found to be involved in various metabolism-related processes, such as pyruvate, sphingolipid, glyoxylate, and dicarboxylate metabolism, as indicated by pathway analysis.

Furthermore, significant enrichment was observed in neuroactive ligand–receptor interaction pathways, cytokine–cytokine receptor interaction, apelin signaling pathway, CAMs, and progesterone-mediated oocyte maturation (Figure 4C,G and Table S5). The comparison results between these two groups revealed 314 DEGs in the F2 stage, and these genes were significantly enriched in pathways related to steroid hormone biosynthesis and progesterone-mediated oocyte maturation, suggesting their close association with follicle development (Figure 4D,H and Table S6).

It is evident that the neuroactive ligand–receptor interaction pathway is commonly enriched in SWF, SYF, F5, and F2 follicles, while the cytokine–cytokine receptor interaction pathway is commonly enriched in SYF, F5, and F2 follicles of both the H-group and L-group hens’ ovaries.

The relevant upregulated DEGs include *NPY4R*, *PENK*, *CTSG*, *HRH2*, *GAL*, *GABRE*, *AGTR1*, *GLP1R*, *DRD4*, *GRM3*, *XCR1*, and *CCL19*. The relevant downregulated DEGs include *OPRK1*, *GPR35*, *CCKBR*, *PGR2/3*, *NPFFR1*, *NMB*, *OXTR*, *ADM*, *CCK*, *PTGDR*, *AGT*, *CCKBR*, *GRM1*, *MC3R*, *CHRNA7*, *CHRNA1*, *MTNR1A*, *APLNR*, *SSTR5*, *CRHR1*, *VIPR1*, *GABRA4*, *GDF5*, *CSF3R*, *TNFSF4*, *GDF9*, *MSTN*, *IL6R*, *INHBB*, *BMP15*, *IL4*, and *EDAR*, among others.

### 3.5. Characterization of the Critical, Potential DEGs Involved in the Production of Eggs

**Figure 5:**
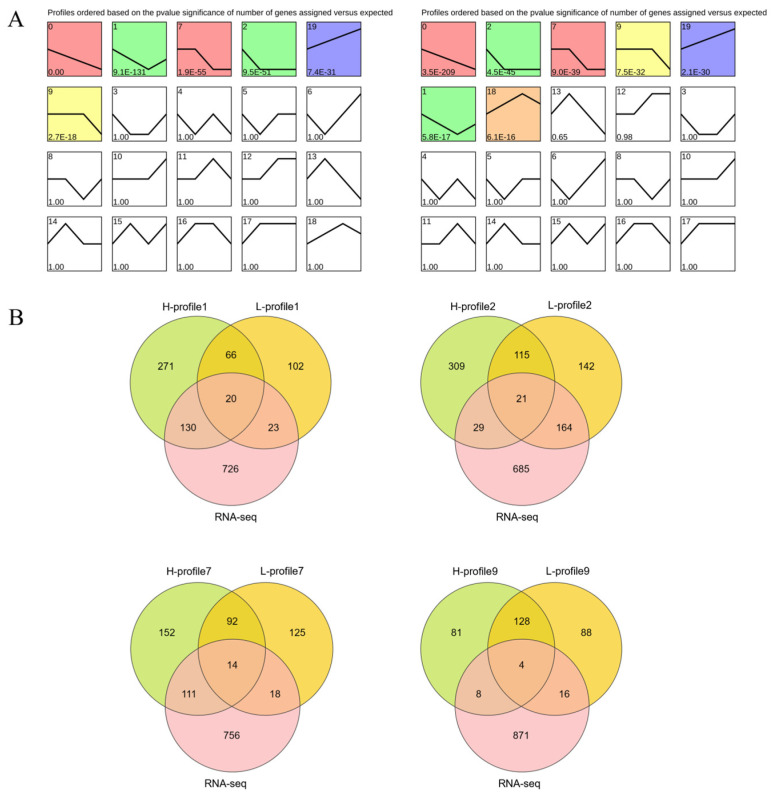
Time-series modules and co-expression of DEGs. (**A**) The left panel shows H-Groups, and the right panel shows L-Groups. The number in the upper left corner indicates the module number. The same color is used to indicate each cluster. The lower left value represents the significance of the enrichment. (**B**) RNA-seq represents the sum of the differential expression of mRNAs for the four developmental stages, and H-profile and L-profile represent the trend analysis modules within the high and low groups, respectively.

We performed a time series analysis using STEM (Short-Term series Expression Miner) to investigate the dynamic expression patterns of protein-coding genes (Figure 5). We discovered 3520 and 2877 DEGs that were grouped into four and five clusters, respectively. These clusters exhibited six and seven significantly enriched patterns (*p* < 0.05, Figure 5A, Tables S7 and S8) based on their dynamic expression patterns across the four developmental stages (Figure 5A). Interestingly, the H-Group and L-Group had similar model profiles that matched the dynamic expression patterns, indicating their comparability in terms of their roles.

We employed co-expression analysis to investigate the functional relationships between DEGs and follicle developmental stages. Firstly, we used DESeq2 to identify DEGs associated with egg production, which included genes showing differential expression across different follicle development stages. Next, Venn diagrams were employed to identify co-expressed genes based on both egg production and follicle expansion. By focusing on modules with changing inflection points and overlapping genes in the dynamic gene expression process, we ultimately identified four notable modules (Figure 5B). Through a comprehensive analysis of these four modules, we found that the 59 overlapping DEGs may play crucial roles in the developmental stages of follicles (Table 3).

### 3.6. Quantitative Real-Time PCR Validation of Gene Expression

**Figure 6:**
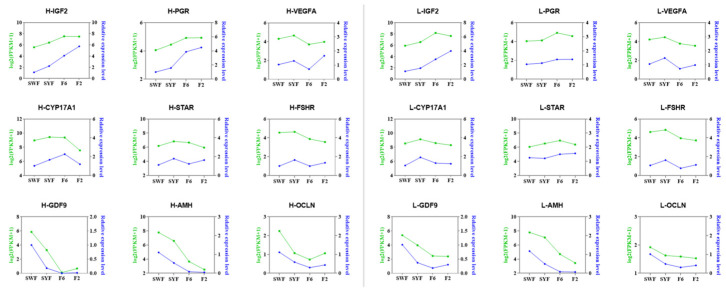
Quantitative validation of nine DEGs in H-Group (**left**) and L-Group (**right**). The green lines are the log2(FPKM + 1) values of RNA-seq, and the blue lines show the relative expression by qPCR.

In these two groups, we randomly chose 9 DEGs (*IGF2*, *PGR*, *VEGFA*, *CYP17A1*, *STAR*, *FSHR*, *GDF9*, *AMH*, and *OCLN*) for qPCR validation (Figure 6). Log2-transformed (FPKM + 1) values, derived from RNA-seq data, were calculated to compare the relative expression levels of the 9 DEGs in each sample.

Except for the *STAR* and *FSHR* genes, the expression patterns of all other genes were consistent between qPCR and RNA-seq. The RNA-seq data supported the altered expression pattern of DEGs across most stages. These results demonstrate our RNA-seq data’s reliability and validate the identified transcripts’ accuracy.

## 4. Discussion

In recent years, there has been an increasing focus on the development and utilization of local chicken breeds in China. However, the analysis of egg production traits, particularly in terms of continuous egg-laying ability, in these local chicken breeds is still limited. Continuous egg production is an important indicator of laying performance in hens and a key target for poultry genetic improvement and breeding management. With the advancements in transcriptomics and the application of high-throughput sequencing technologies, exploring the relationship between the ovarian follicle transcriptome and continuous egg production traits in poultry has become necessary. This exploration aims to enhance our understanding of breeding and reproductive management in laying hens and promote the development of local chicken breeding programs. Therefore, in this study, we assessed the egg-laying capacity of a population through the quality evaluation of continuous egg production. The ovarian follicle transcriptome profiles of hens with different egg production traits were determined at four key developmental stages (i.e., SWF, SYF, F5, and F2) using high-throughput sequencing technology. The objective of this study is to gain a deeper understanding of the regulatory mechanisms underlying the differences in follicle development and maturation between these two groups of hens.

We attempted to explore the DEGs in the SWF, SYF, F5, and F2 follicles between the H-Group and L-Group of Taihang chickens. This approach is important for investigating potential key genes and pathways associated with continuous egg-laying traits at different developmental stages of hen ovarian follicles. Through further enrichment and analysis of DEGs, we discovered that follicles at different developmental stages possess their unique molecular genetic features and play distinct roles in promoting ovarian growth and development. DEGs in SWF follicles were significantly enriched in pathways related to immune response, adhesion, and phosphatidylcholine metabolism. DEGs in SYF were significantly enriched in pathways related to stress response and monocarboxylic acid transport. Prehierarchical follicles (F5 and F2) were primarily associated with pathways involving lipid metabolism, cholesterol homeostasis, and ion transport. These results are consistent with the physiological processes of follicle growth. The study indicates that these pathways and functional genes are crucial in regulating follicle development. For example, the exogenous addition of Lysophosphatidylcholine can alleviate the inhibition and damage of zearalenone (ZEA) in the maturation process of oocytes [13].

Many growth factors and cytokines play critical roles in follicle development and regulation. For example, *GDF-9* is essential for ovarian folliculogenesis. Female mice lacking *GDF-9* can still form primordial and primary follicles, but it affects their follicle development, resulting in infertility [14]. This observation holds true in human ovaries as well [15].

Gonadotropin hormones and intrafollicular factors determine whether a follicle will continue developing or entering the atresia pathway [16]. *IGF*, *TGFβ*, and *BMP* synergistically interact with *FSH* in a coordinated manner during follicle growth and development [16,17,18]. The *FSHR* and *AMH* play crucial roles in maintaining the activity of prehierarchical follicles and in the selection and grading of follicles [19,20,21].

In Low-continual-producing Taihang hens, the mRNA levels of *GAL*, *COL10A1*, and *RAD9A* were lower in prehierarchical follicles (SWF and SYF), while the mRNA levels of *FAM83A*, *GDF5*, *CHAT*, and *MMP1* were higher. Before follicle selection, granulosa cells exhibit active mitotic activity and remain undifferentiated [22], thereby increasing the number of follicles. Oogonia undergo gradual meiotic division based on mitotic division. One of the representative genes, *RAD9A*, is a DNA integrity checkpoint control system component and plays an important role in DNA repair. *RAD9A* is involved in the DNA integrity checkpoint control system and plays a critical role in DNA repair, an important aspect of the typical process of meiotic division. Previous studies have demonstrated that *RAD9A* is crucial in efficient DNA damage repair during mammalian meiotic division, which is essential for reproductive competence [23]. The absence of *RAD9A* in mice results in complete sterility due to impaired DNA damage repair function [24]. Although the exact role of *RAD9A* in chicken ovarian follicle function is not yet clear, studies have indicated that mutations in a class of genes involved in the repair of double-stranded DNA breaks caused by recombination during oocyte development, can activate the meiotic checkpoint, thereby disrupting oocyte development [25,26]. Therefore, it can be speculated that the downregulation of *RAD9A* transcripts may lead to abnormal follicle development.

During the prehierarchical follicle stage, follicles do not exhibit hormone activity. However, once a follicle is selected, granulosa cells can rapidly produce small amounts of progesterone [22]. Conversely, if the follicle is not selected, it will undergo atresia. After follicle selection into the prehierarchical stage, the *FSHR* induces progesterone production, activates the cAMP signaling pathway, and promotes the expression of downstream steroid-related genes such as *LHR*, *StAR*, *CYP11A*, and *CYP19A1* [27,28]. During this stage, blood vessels appear on the surface of the follicle, providing more nutrients for the rapid deposition of yolk substances within the selected follicle and promoting follicle growth. Our research findings indicate significant enrichment of pathways involving progesterone-mediated oocyte maturation, steroid hormone biosynthesis, and ECM-receptor interactions at this stage. The growth and development of follicles require the expression of the extracellular matrix (ECM) [4,29], with collagen being its main component. The ECM plays a crucial role in supporting the structural integrity of the follicle during follicle growth and yolk deposition processes. A study conducted by Sun et al. on the follicles of 51-week-old Nandan Yao chickens further confirms the key role of collagen in maintaining follicle morphology during follicle growth [4], providing additional support for the crucial involvement of the ECM in follicular development.

Trend clustering analysis was performed using STEM on two groups of differentially expressed mRNAs. Venn diagrams were used to identify genes that coexisted within the same module of DEGs and between the two groups. Potential key mRNAs were studied based on trend clustering analysis, and their functions can explain the observed changes in expression levels at the corresponding stages. The featured genes include *KCNH1*, *IGFs* [30,31], *HMGA2*, and *CDH1*. Although the functions of *KCNH1*, *HMGA2*, and *CDH1* in chicken ovarian follicles have not been studied, research suggests that the absence of *KCNH* channels can affect the function of the mouse uterine smooth muscle layer, and their functional impacts may be determined by changes in *KCNH* gene expression [32,33]. Increasing evidence suggests that the insulin-like growth factor system plays a role in various physiological processes related to female reproduction, such as oocyte development [34] and folliculogenesis [35,36]. *HMGA2* regulates angiogenesis through *IGF2BP2* induction [37]. In mice, the loss of both alleles of *HMGA2* leads to infertility [38]. Additionally, the overexpression of *HMGA2* promotes faster myoblast proliferation and higher vascularization, indicating its important role in regulating and activating angiogenesis in uterine smooth muscle cells [39]. Our data suggests that the downregulation of *HMGA2* in SYF promotes follicle selection. In comparison to the low group, the high expression of *HMGA2* allows follicles to continue their development, resulting in a longer time spent in the hierarchical follicle development stage. This ultimately leads to differences in continuous egg-laying traits between the high and low groups. Phenotypic studies on high-and-low-egg-producing Jinding ducks [5] revealed a significant difference in oviduct length between the two groups (*p* < 0.05). However, the two groups had no statistically significant differences in body weight, pre-ovulation follicle count, SYF, and LWF counts (*p* > 0.05). These findings provide theoretical support for explaining variations in egg production among poultry, suggesting that the time required for follicles to grow and mature may be an important influencing factor rather than the number of follicles. *CDH1* is involved in maintaining adhesive connections between follicular cells and regulates cell proliferation, differentiation, and arrangement within the follicle. It plays a critical role in follicle morphology through dynamic interactions with the actin cytoskeleton, mediating cell adhesion. *CDH1* participates in maintaining adhesive connections between follicular cells and regulates cell proliferation, differentiation, and arrangement within the follicle. It plays a crucial role in follicular morphology because *CDH1* mediates intercellular adhesion through dynamic interactions with the actin cytoskeleton [40,41]. Mice with *CDH1* abnormalities exhibit reproductive defects [42]. However, transcriptomic results only provide preliminary clues, and further validation and functional studies are needed to confirm the significance and mechanisms of these findings.

## 5. Conclusions

This study identified a total of 4253 DEGs by selecting hens with different continuous egg-laying abilities. These genes may play important roles in regulating ovarian follicle development in chickens. The data describe the complex mechanisms that regulate follicle growth and development, including connectivity, phagocytosis, immunity, and metabolism. Additionally, through joint STEM analysis, potential key roles of *KCNH1*, *HMGA2*, and *CDH1* in the dynamic development of follicles were identified. These findings lay the foundation for understanding follicle development and egg-laying traits in laying hens.

## Figures and Tables

**Table 1:** Gene primer sequences.

Primer Name	Primer Sequences	Product Length
*VEGFA*-F	ATATTCAGGCCATCCTGTGTGC	159
*VEGFA*-R	CTGTAAGAAGCTCATGTGCGCT	
*GDF9*-F	TCTATTGCTGCCTTCACTGGCT	196
*GDF9*-R	TGGTCAGACAGCACTTCGAGCA	
*IGF2*-F	ATGTGTGCTGCCAGGCAGATAC	198
*IGF2*-R	AATGCCACGGTTGATCCTCCTG	
*CYP17A1*-F	ACTACGTGGTGGTGGTCAACAG	205
*CYP17A1*-R	GAGCCCTCCCCAAACATGGAGA	
*STAR*-F	AAGCCCTTCAGCGAGATGGAGA	189
*STAR*-R	CTGATCCACCACCACCTCCAGG	
*FSHR*-F	ATGTCCTTGGGTCTCACCTGC	204
*FSHR*-R	TCCTGTGAAAGCTCCCTTCGG	
*AMH*-F	GGAGGAGATGGGACTTGGAGC	204
*AMH*-R	TCGTTGCGGTCCATCTTCAGG	
*PGR*-F	ATGACCGAGGTGAAGAGCAAGG	184
*PGR*-R	CCTCCTCCTCCTCCTCGTTCT	
*OCLN*-F	CTGGCCTTCGTCATGCTCATC	208
*OCLN*-R	TGACGATGAGGAACCCACAGAC	
*GAPDH*-F	GAACATCATCCCAGCGTCCA	132
*GAPDH*-R	CGGCAGGTCAGGTCAACAAC	

**Table 2:** Descriptive statistics of differentially expressed genes in high and low groups.

Group	Up	Down	All
LSWF vs. HSWF	58	38	96
LSYF vs. HSYF	32	167	199
LF5 vs. HF5	51	541	591
LF2 vs. HF2	33	281	314

Small white follicles in the low continual production group (LSWF), Small white follicles in the high continual production group (HSWF), Small yellow follicles in the low continual production group (LSYF), Small yellow follicles in the high continual production group (HSYF), Small yellow follicles in the low continual production group (LSYF), Small yellow follicles in the high continual production group (HSYF), F5 follicles in the low continual production group (LF5), F5 follicles in the high continual production group (HF5), F2 follicles in the low continual production group (LF2), and F2 follicles in the high continual production group (HF2).

**Table 3:** The 59 overlapping DEGs.

H-Profile1 vs. L-Profile1 vs. RNA-seq	H-Profile2 vs. L-Profile2 vs. RNA-seq	H-Profile7 vs. L-Profile7 vs. RNA-seq	H-Profile9 vs. L-Profile9 vs. RNA-seq
*PKP1*	*SEMA6B*	*MYL2*	*CXCL14*
*TNN*	*RBM44*	*KCNK2*	*KERA*
*HPRTL*	*SNAP47*	*CDH1*	*CYP19A1*
*ZP4*	*PIK3AP1*	*RAB11FIP4*	*SUV39H1*
*MYT1*	*DNAH12*	*VWA2*	
*AGBL1*	*MMP11*	*HMGA2*	
*BCAS1*	*SHROOM1*	*VLDLR*	
*XDH*	*TMEM62*	*PRKCH*	
*KCNH1*	*LRRC34*	*CCNA1*	
*TBPL2*	*SGMS2*	*MYCL*	
*MFNG*	*CHGA*	*ENSGALG00000027090*	
*AKR1D1*	*SGIP1*	*VPS25*	
*UPK1B*	*GPR137C*	*LIPG*	
*CD80*	*TERT*	*FAM71D*	
*SLC22A3*	*JAKMIP1*		
*ENSGALG00000024136*	*GJB6*		
*CXCL13L3*	*GJB2*		
*C2H8ORF22*	*ATAD3A*		
*RNF17*	*FHDC1*		
*SLC4A5*	*IGF2BP1*		
	*BCDIN3D*		

Profile1 of STEM in the high continual production group (H-profile1), Profile1 of STEM in the low continual production group (L-profile1), Profile2 of STEM in the high continual production group (H-profile2), Profile2 of STEM in the low continual production group (L-profile2), Profile7 of STEM in the high continual production group (H-profile7), Profile7 of STEM in the low continual production group (L-profile7), Profile9 of STEM in the high continual production group (H-profile9), Profile9 of STEM in the low continual production group (L-profile9), all the differential genes between the high-yield group and the low-yield group were compared between the four periods (RNA-seq).

## Data Availability

All data in this study are included in the article content and the Supplementary File. Transcriptome data were uploaded to the NCBI database sequence read archive, accession number PRJNA1045970.

## Supplementary Materials

The following supporting information can be downloaded at https://www.mdpi.com/article/10.3390/genes15010040/s1. Table S1: Quality analysis and mapping of RNA-Seq data to the chicken reference genome; Table S2: Differentially expressed mRNAs in pairwise stages; Table S3: List of DEGs and functional enrichment analysis results for DEGs at the stage of SWF; Table S4: List of DEGs and functional enrichment analysis results for DEGs at the stage of SYF; Table S5: List of DEGs and functional enrichment analysis results for DEGs at the stage of F5; Table S6: List of DEGs and functional enrichment analysis results for DEGs at the stage of F2; Table S7: List of H-Group mRNAs in STEM map of significantly different modules; Table S8: List of L-Group mRNAs in STEM map of significantly different modules.

## Abbreviations

*GDF9*—growth differentiation factor-9; *IGF*—Insulin-like growth factor; *TGF-β*—transforming growth factor-β; *BMP*—bone morphogenetic protein; *FSH*—follicle-stimulating hormone; *FSHR*—follicle-stimulating hormone receptor; *AMH*—anti-Müllerian hormone.
